# Goldilocks and the SOS pathway: Finding “just right” for growth after salt stress

**DOI:** 10.1093/plcell/koaf118

**Published:** 2025-07-01

**Authors:** Rory Osborne

**Affiliations:** Assistant Features Editor, The Plant Cell, American Society of Plant Biologists; School of Biosciences, University of Birmingham, Birmingham B13 2TT, UK

As the story goes, Goldilocks had the luxury of choosing her ideal porridge, chair, and bed, before fleeing the bears’ house. Plants, however, must endure whatever conditions they encounter, whether they are or are not “just right.” And unlike Goldilocks, who lived in a fairytale, plants face escalating pressure as climate change pushes environmental conditions to further extremes.

Adaptation to a toxic overaccumulation of Na^+^ involves the SALT OVERLY INSENSITIVE (SOS) pathway. High concentrations of Na^+^ activate membrane-bound calcium channels that import Ca^2+^ to the cytoplasm. This influx is perceived by calcineurin B-like (CBL) and CBL-interacting kinases (CIPK), which phosphorylate SOS2 and SOS3. These then coactivate the membrane-bound Na^+^/H^+^ antiporter SOS1, which facilitates Na^+^ export to the apoplast (Halfter et al. 2000; Zhu 2002). While the SOS pathway itself has been well characterized, it remains unclear how the balance between growth and salt stress response is maintained. This prompted **Kun-Lun Li, Hui Xue, and colleagues (Li et al. 2025)** to investigate growth arrest in *Arabidopsis* in elevated salinity.

Previous work from the same group showed that K^+^ levels affected the master regulator of growth, TARGET OF RAPAMYCIN COMPLEX (TORC; Li et al. 2023). Hypothesizing that the same would be true for Na^+^, their investigation began by quantifying the phosphorylation of the TORC substrate RIBOSOMAL S6 KINASE 1 (S6K1). Phosphorylated S6K1 (P-S6K1) levels rapidly decreased in response to NaCl, independently of the stress hormone ABA, suggesting a direct relationship between TORC activity and Na^+^ levels.

Next, by evaluating the interactions of components of the SOS pathway and TORC, the authors showed a direct physical interaction between CIPK24 (a regulator of SOS2) and RAPTOR1B (the major isoform of the RAPTOR subunit of TORC, see Figure). Critically, CIPK24 conditionally phosphorylated RAPTOR1B at Ser897 in response to NaCl treatment. Phosphorylation of RAPTOR1B at this residue promotes its disassociation, and thus inhibition, of TORC. Conversely, TORC activity increased in a *cipk24* knockout mutant, in which the salt-induced decrease in P-S6K1 levels was attenuated. Increased TORC activity in *cipk24* was further evidenced by seedling recovery after NaCl treatment. Five days after treatment, wild type and RAPTOR1B mutant (*raptor1*) roots grew by ∼200%, while *cipk24* mutant roots grew by over 600%. Remarkably, the increased rate of recovery in *cipk24* was completely abolished by a *raptor1* mutation or when TORC was inhibited by the selective TOR inhibitor AZD8055. Taken together, these results suggested that salt-induced growth attenuation requires NaCl-dependent and CIPK24-mediated repression of TORC.

**Figure.**
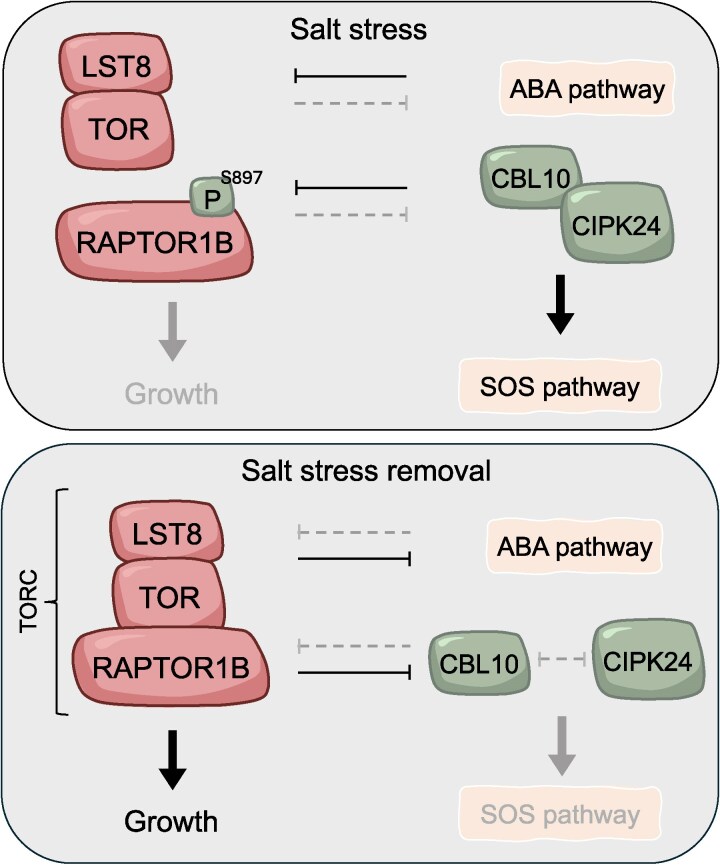
Model proposed by Li and colleagues, describing the role of TORC in regulating the transition from salt stress conditions (top) to normal conditions (bottom). Adapted from Li et al. (2025), Figure 6.

Having described the regulation of TORC during “too salty” conditions, the authors next considered what reciprocal role TORC might have on the SOS pathway during “just right” conditions—particularly as the CBL10-CIPK24 module is deactivated during the recovery from salt stress (Li et al. 2020). Acting upstream, CBL10 directly phosphorylates CIPK24. By quantifying phosphorylated CLB10 (P-CBL10) levels as a proxy for CIPK24 activity, the authors showed a clear reduction of P-CBL10 during salt recovery, which could be blocked by inhibiting TORC.

Finally, coexpression and immunoprecipitation analysis of CBL10/CIPK24 with or without RAPTOR1B and NaCl revealed a competitive relationship between these 3 proteins. Under high salt (“too salty”) conditions, the CBL10/CIPK24 interaction is strengthened, promoting CIPK24 phosphorylation, TORC inhibition, and growth repression. During recovery (“just right”) conditions, RAPTOR1B disrupts the CBL10/CIPK24 interaction, blocking the phosphorylation of CIPK24 and its downstream repressive effect on growth. Thus, the authors reveal an ABA-independent mechanism regulating plant response to salt stress and provide further evidence that the TOR complex acts as a focal point in balancing growth and stress trade-offs in plants.

## Recent related articles in *The Plant Cell*

- Ma et al. (2023) describe a phytochrome-dependent mechanism that supports the SOS pathway; photoactive PhyA and PhyB physically interact with SOS2 to promote its kinase activity under salt stress in the light.
- Park et al. (2023) show that early flowering in response to salt stress requires the post-translational regulation of GIGANTEA by SOS3, creating a molecular switch that integrates multiple signals under Na^+^ that regulates *CONSTANS* expression.
- Liu et al. (2025) used cell biology and confocal imaging techniques to demonstrate the vacuole of root cells fragments in response to salt. This adaptive response increases the vacuolar surface-to-volume ratio, facilitating stronger Na^+^ sequestration.
- Ma et al. (2025) describe a novel mechanism that regulates plant physiology under salt stress and variable nutrient composition, mediated through the interaction of SOS2 and AMMONIUM TRANSPORTER1;1 (AMT1).

## Dive Curated Terms

The following phenotypic, genotypic, and functional terms are of significance to the work described in this paper:

SOS3 Gramene: AT5G24270
SOS3 Araport: AT5G24270

CBL Gramene: AT3G57050
CBL Araport: AT3G57050

CBL10 Gramene: AT4G33000
CBL10 Araport: AT4G33000

SOS1 Gramene: AT2G01980
SOS1 Araport: AT2G01980

RAPTOR1 Gramene: AT3G08850
RAPTOR1 Araport: AT3G08850

SOS2 Gramene: AT5G35410
SOS2 Araport: AT5G35410

Gigantea Gramene: BRADI2G05226
Gigantea Araport: BRADI2G05226
